# Distress among Korean Cancer Survivors: A Latent Profile Analysis

**DOI:** 10.3390/ijerph19031613

**Published:** 2022-01-30

**Authors:** Kwang-Hi Park, Min Kyung Song

**Affiliations:** 1 College of Nursing, Gachon University, Incheon 21936, Korea; parkkh@gachon.ac.kr; 2 Department of Nursing, College of Medicine, University of Ulsan, Ulsan 44610, Korea

**Keywords:** cancer survivors, distress, latent profile analysis

## Abstract

This study aimed to classify cancer survivors’ latent profile analysis (LPA) according to the problem list and identify the differences in distress between subgroups. Furthermore, this study identified differences between subgroups based on their demographic and clinical characteristics. A self-reported cross-sectional survey was administered to 446 adult cancer survivors in Korea. A distress thermometer and problem list were used, and four domains of the problem list were used to perform LPA and create subgroups. Quade’s non-parametric analysis of covariance was used to determine the difference in distress between the profiles. The three identified subgroups of the problem list were: “low problem group” (36.7%), “high problem group” (49.1%), and “family only low problem group” (14.2%). The analysis showed that there was a difference in the distress level according to the sub-profile of the problem list (F = 43.69, *p* < 0.001). In interventions for distress, integrative interventions that are not limited to one area are necessary, and cultural characteristics as well as the problem list relevant to cancer survivors should be considered.

## 1. Introduction

Cancer is a leading cause of death worldwide [1]. Advances in early diagnosis and treatment technologies for cancer have significantly extended the life expectancy of cancer patients, and the number of cancer survivors is rapidly increasing [2]. In Korea, the number of cancer patients who survived more than five years after being diagnosed with cancer was about 1.16 million in 2018 [3], that is, 1 in 45 people in the total population. Therefore, concerns for their health problems are also increasing.

Cancer survivors who have completed life-threatening surgery and chemotherapy can generally return to their previous lives in a healthy state. However, they may suffer from later treatment-related side effects, including concerns regarding cancer recurrence and the development of new malignancies, along with various physical and psychosocial problems due to disease or treatment [4].

The National Comprehensive Cancer Network (NCCN) introduced the concept of distress, recognizing it as an important aftereffect of cancer diagnosis and treatment. Distress is defined as any emotional experience that is psychologically, socially, or spiritually unpleasurable, including any cognition, behavior, and emotion that can reduce one’s level of adaptation to cancer and cancer-related symptoms and treatment [5]. The most common problems cancer survivors experience are fatigue, pain, depression, sleep disorders, and anxiety [6,7]. This study developed and used a distress thermometer and problem list in research and clinical settings as tools to assess distress of cancer survivors.

A previous study reported that at least one-third of long-term survivors experienced a clinically high level of distress within five years of the end of their treatment [8]. Furthermore, compared to the general population, the distress of cancer survivors was high [9,10], and survivors experience a level of distress that is similar to cancer patients undergoing treatment [10]. In a study on breast cancer survivors, 63.1% of cancer survivors showed severe or more than severe distress; furthermore, emotional domains in the problem list, including anxiety, fear, sadness, and depression, accounted for the largest number [11].

Although the distress levels of cancer survivors and the importance of managing the problem list have been recognized, most past studies have not confirmed the degree of their distress by examining the problem list using yes-or-no questions, and it is not possible to determine whether the problem list can be clustered. Furthermore, although these problems rarely occur alone, they are often identified and managed independently. If problems that occur simultaneously are analyzed by grouping them together rather than managing them independently, more useful information can be provided for symptom management [12]. Latent Profile Analysis (LPA) is a person-centered statistical technique that assumes that the average pattern of an observed variable can be explained by an individual’s unique latent class or subgroup. This approach is useful for identifying the characteristics of cancer survivors’ problem lists and explaining how they are translated into distress outcomes. In addition to identifying these subgroups, it is also important to assess certain demographic and clinical characteristics, which will lead to the development of various tailored interventions.

This study aims to identify the problem list profile of cancer survivors using LPA and examines the demographic and clinical characteristics related to this profile. Furthermore, this study investigates the relationship between the problem list profile and distress.

## 2. Materials and Methods

### 2.1. Study Design and Participants

The cross-sectional exploratory study using LPA for the problem list of cancer survivors was performed by four domains of problem list, and differences in distress between profiles were identified. The participants of this study were adult cancer survivors aged 18 years or older who had completed the active treatment (surgery, standard chemotherapy, and radiation therapy) within 5 years. Recruitment was conducted between May and June 2021. To voluntarily recruit participants, leaflets were posted on the social media of the nation cancer survivor support centers (National Cancer Center Korea, three regional centers) and the Cancer Education Center of two hospitals in Seoul. Inclusion criteria were 18 years of age or older, diagnosed with one cancer, the end of major treatment including surgery, chemotherapy, and radiation therapy within 5 years, and no cancer at present.

### 2.2. Ethical Approval

This study was conducted under the ethical approval of the Institutional Review Board. All the respondents participated in this study voluntarily and anonymously. They were aware of the purpose and duration of the study and that they could withdraw from the study at any time.

### 2.3. Measures and Data Collection

#### 2.3.1. Questionnaire

The questionnaire included items on demographic characteristics (age, gender, educational level, marital status, major health caregiver, employment status, income, residence), clinical characteristics (cancer types, time of diagnosis, period since last treatment), subjective health status, and the distress thermometer and problem list. Content validity of the questionnaire was evaluated among a group of 11 professionals (3 doctors, 4 nurses, 4 nursing professors). Experts were able to make recommendations on the questionnaire items. The content validity index of the questionnaire items was 0.8 or higher, and all items were maintained [13]. The contents of some items were supplemented to increase the understanding of the subjects of the questionnaire and to facilitate the flow of the sentences. Subjective health status was measured on a 4-point Likert scale ranging from very unhealthy (1 point) to very healthy (4 point) with one question asking the participant’s current health status.

The Distress Thermometer and Problem List (DT & PL). Distress was measured using the Korean distress thermometer (DT) and problem list (PL), which were developed by NCCN in the US and have established validity in Korean cancer patients [14]. In the first step, participants are asked to select the number that best represents the degree of psychological distress they have experienced over the past week on an 11-point visual analog scale ranging from 0 (no distress) to 10 (extreme distress). A higher score indicates a higher level of distress. In the second step, the participant checks the items that have been a problem affecting their distress over the past week. The list of problems has been revised into 20 items (depression/sadness, fear/worry, nervousness/irritability, loss of motivation, change in appearance (hair loss, skin color, and so on), diet (weight/intake change), fatigue, indigestion (nausea), memory/reduced concentration, pain, sleep disorder, numbness of limbs, anxiety about recurrence/death, worries about the meaning of life, raising children, economic problems, work/school, problems with children, relationship with spouse, and relationship with parents) presented by the National Cancer Information Center [15] and each item was measured on a scale of 0 (no)–10 (extreme). The reliability (Cronbach Alpha) in this study was 0.92.

#### 2.3.2. Data Collection

Data collection was conducted through an online survey (google forms) from 25 May to 10 June 2021. At the beginning of the online survey, participants read an information sheet and filled out informed consent, along with guidance on inclusion and exclusion criteria. Participants were guaranteed the right and privacy to voluntarily participate in the study. To avoid missing data, all questions in the online survey were set so that respondents had to answer a question before advancing to the next question. Survey completion times ranged from 15 to 25 min, with an average of about 20 min. The final number of participants in the questionnaire was 442, of which 6 duplicate participants, 33 people who did not meet the criteria (currently on treatment, survivors over 5 years), and 401 people excluding 2 insincere responses were used for the analysis.

### 2.4. Statistical Analysis

This study performed an exploratory factor analysis by inputting 20 problem lists. Principal component analysis was used as a factor extraction method, and Varimax was used as a rotation method. Based on the analysis, the relationship with parents, where the factor loading was less than 0.5, was deleted. As per the final analysis, measurement items were loaded into the following four indices: (1) physical problems (appearance change [hair loss, skin color, and so on], diet [weight/intake change], fatigue, indigestion [nausea], memory/reduced concentration, pain, sleep disorder, numbness of limbs); (2) emotional problems (depression/sadness, fear/worry, nervousness/irritability, lack of motivation, anxiety about recurrence/death, worry about the meaning of life); (3) functional problems (economic problems, work/school); and (4) family problems (problems with children, relationships with spouses, raising children). Using the four indices, LPA was performed to identify the types of the latent profiles in the problem list. To determine the appropriate number of class solutions, we considered the fit index, statistical significance, and entropy index [16]. Bayesian information criterion (BIC) and Sample size adjusted BIC (saBIC) were used as fit indices, and Lo-Mendell-Rubin likelihood-ratio test (LMR) and the bootstrap likelihood-ratio test (BLRT) were used as statistical significance test methods. A smaller BIC and saBIC indicates greater suitability in the model. LMR and BLRT are goodness-of-fit indices that compare the hierarchical results through statistical power. If the *p* value is significant when K classes and K-1 classes are compared, the goodness-of-fit that includes additional class is better [17]. For entropy, a value of 0.80 or higher indicates appropriate classification accuracy [18]. A chi-square test and one-way analysis of variance (ANOVA) were used to investigate whether demographic and clinical characteristics differed between classes. To analyze the differences in distress levels according to the identified problem list types, Quade’s non-parametric analysis of covariance (ANCOVA) was performed by controlling sociodemographic variables.

## 3. Results

### 3.1. Participant Characteristics

The average age of the participants was 45.74 ± 8.14 years, and 51.4% of participants were in their 40s. Three hundred seventy-six (93.8%) were female and twenty five (6.2%) were male. Most of the participants (*n* = 283, 70.6%) graduated from college. As for marital status, 291 (72.6%) were married, and the main caregiver was a spouse or themselves (37.7% or 32.4%, respectively); 64.3% were unemployed, and the monthly household income was 2–4.99 million won for 42.9% of the participants and more than 5 million won for 42.1% of the participants. Further, 290 participants (72.3%) were living in a metropolitan area. For clinical characteristics, we assessed the type of cancer, the time when the cancer was first diagnosed, the period after the last treatment was completed, and the subjective health status. The diagnosis was breast cancer in 254 cases (63.3%), gynecological cancer in 40 cases (10.0%), thyroid cancer in 33 cases (8.2%), stomach cancer in 31 cases (7.7%), and others such as colorectal cancer, kidney cancer, lung cancer, hematologic malignancy, liver cancer, pancreatic cancer, head and neck cancer, and prostate cancer. Among the study participants, 62.3% were diagnosed with cancer for over two years and within five years. The duration after the end of the last treatment was mostly “less than a year” for 141 patients (35.2%) and ranged from “one year or more” to “less than two years” for 120 patients (29.9%). The subjective health status of the study participants was 2.69 ± 0.61 points out of four (Table 1).

### 3.2. Problem List Profile Analysis for Cancer Survivors

**Figure 1:**
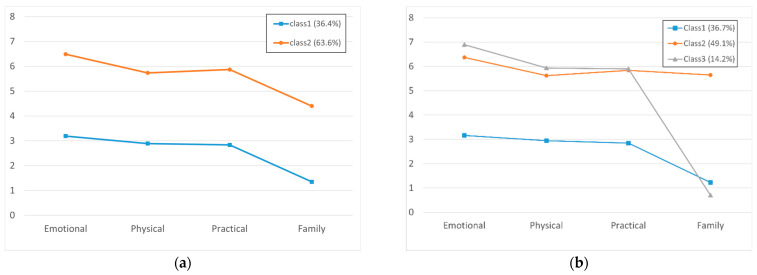
Profile solution: (**a**) 2-profile solution; (**b**) 3-profile solution.

To explore and analyze the problem list profile of cancer survivors, we determined the number of latent strata by applying LPA and confirmed the characteristics of each group of the determined latent strata. The indices inputted into the analysis model were four domains (emotional, physical, functional, and family). As a result of exploring the cancer survivors’ problem list profile type, models with one to four classes were measured (Table 2). The number of groups was finally determined based on the model fit indices and the latent class distribution rate. BIC and saBIC decreased as the number of classes increased and were lowest in Model 4. Entropy was closest to 1 in Group 3. LMR and BLRT were statistically significant in Models 2 and 3, confirming the heterogeneous groups in the problem list. Profile Model 3 had lower BIC and saBIC than Model 2, and LMR and BLRT showed that both models supported the goodness-of-fit that includes additional class is better. Models 2 and 3 both confirmed that the information and latent class distribution rate were more than 5%. Figure 1 provides a visual depiction of Models 2 and 3. Model 3 has more information than Model 2, and its classification rate was from 14.2% to 49.1%, indicating high practicality. Consequentially, Model 3 was finally selected for further analysis. The average latent class probabilities of Model 3 ranged from 0.896 to 0.960, close to 1.0, confirming that the classification accuracy was high [19].

**Figure 2:**
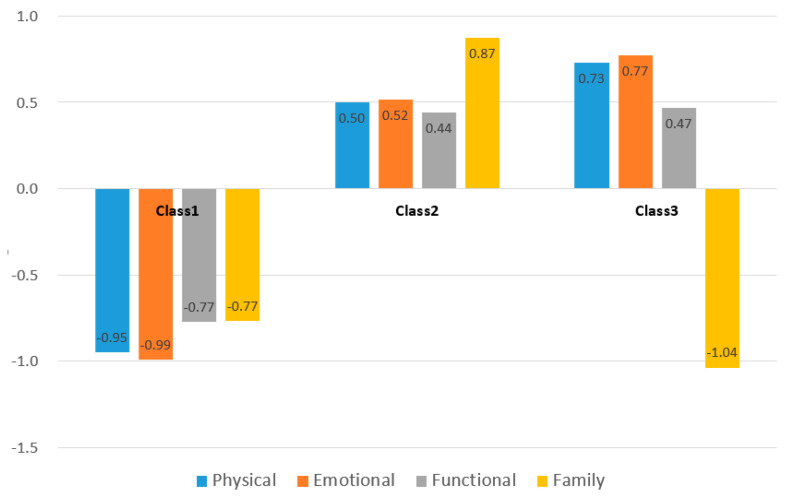
Illustration of z-score distribution of problem list in the three profiles defined in the latent profile analysis.

Table 3 shows the cancer survivor problem list profile derived from the four indices. The explored latent classes were named by comparing and observing the changing patterns of the four indices. Class 1 (36.7%, *n* = 147) was a “low problem group”, and physical, emotional, functional, and family problems were low. Meanwhile, Class 2 (49.1%, *n* = 197) was a “high problem group”, and emotional, physical, functional, and family problems were high. Class 3 (14.2%, *n* = 57) is a “group with only low family problems.” Emotional, physical, and functional problems were high, while family problems were the lowest (Figure 2). Looking at each sub-problem, the anxiety about recurrence/death, worry about the meaning of life, and fatigue were the highest among all participants at 5.92 ± 2.68, 5.88 ± 2.70, and 5.84 ± 2.33, respectively. This pattern appears similarly in Classes 1 through 3.

### 3.3. Distress According to the Identified Latent Classes

The average distress score of all the cancer survivors was 4.97 ± 2.17. Controlling demographic and clinical variables, we found significant differences in distress among groups in the problem list profile. Class 1 showed the lowest distress, followed by Class 2 and Class 3. Due to pairwise comparisons of groups analysis, there was a statistically significant difference among Classes 1, 2, and 3, but there was no statistically significant difference in distress between Class 2 and Class 3 (Table 4).

### 3.4. Demographic and Clinical Characteristics across Identified Classes

Table 5 summarizes cancer survivors’ demographic and clinical characteristics and shows the differences of characteristics between subgroups. According to the problem list profile group, there were statistically significant differences in age, education level, marital status, primary caregiver, monthly income, cancer type, time of diagnosis of cancer, period since last treatment, and subjective health (Table 5).

## 4. Discussion

This study identified subgroups of the problem list of cancer survivors by using LPA and its association with distress. Due to the analysis, three distinct profiles were identified for cancer survivors according to the average of four indices (physical, emotional, functional, family). In Class 1, the average of all four problems was low; in Class 2, the average of all four problems was high; and in Class 3, the average of all three problems was high except for family problems. In this study, Class 1 with a high level of all four problems was the most prominent (49.1%). It was confirmed again that a high proportion of cancer survivors who had finished treatment experienced various problems [8].

As for the problems experienced by the participants, emotional problems received the highest score at 5.28 ± 2.16, followed by functional, physical, and family problems. This was similar to a previous study reporting that 33% of cancer survivors experienced pathological emotional distress, and 70% experienced depression, anxiety, fear, and so on [20,21]. The scores of the four indices for all participants seemed to be different, but an examination of each class showed that the degree of physical, emotional, and functional problems (except for the family problem) appeared to be the same. In short, the three problems were either high or low simultaneously. Cancer survivors receive regular medical follow-up after completing treatment, but most medical follow-ups focus only on relapse, medical problems, and physical symptoms, thus excluding psychological symptoms. As psychological health and physical health are interrelated and have a complex relationship [22,23], instead of merely dealing with specific symptoms or problems, it is important to develop an integrated support program for follow-up management.

The cancer survivors who participated in the study had an average distress score of 4.97, higher than the cutoff point of 4, even though they had finished treatment [14]. An examination of each class showed that Class 1, a low problem group, had the lowest distress score of 3.33; Class 2, a high problem group, had the higher distress score of 5.81; and Class 3, a group with only a low family problem, had the highest distress score of 6.28. The distress score was higher as participants experienced more problems. Still, the distress score was highest in Class 3, where there were few family problems, but other physical, emotional, and functional problems were relatively higher than in other groups. Further examinations must be conducted on the characteristics of the participants.

In our study, among demographic characteristics, age, marital status, primary caregiver, educational level, and monthly income differed according to class. In Class 1, those in their 40s or younger accounted for 65.5%, while Classes 2 and 3 accounted for 81.7% and 89.0%, respectively, and the average age was the lowest in Class 3. This was consistent with a symptom cluster study on breast cancer patients, which found that cancer survivors who experienced a higher symptom cluster were younger than asymptomatic ones [24,25,26]. These age-related differences are thought to be associated with greater adverse events and long-term late side effects, given that younger patients often have more invasive forms of cancer and receive more aggressive cancer treatments [27]. Furthermore, younger survivors may have higher expectations for the resumption of family and social roles, and these expectations may lead them to experience higher distress about symptoms [28]. Therefore, health care providers need to pay more attention to young patients while managing the distress of cancer survivors. The proportion of married participants was high at 89.8% in Class 2, but 68.4% were unmarried in Class 3. However, unlike the results of previous studies, which reported no significant difference in distress according to marital status in similar age groups [29], this study noted a difference in distress according to the age of the subjects. Future research is necessary for confirming this difference in distress, that is, whether it arises due to age or marital status. As for the primary caregiver, self-care was the most common in Class 1 with 46.9%, the spouse was the most common in Class 2 with 43.1%, and the parent or sibling was the most common in Class 3 with 49.1%. A previous study on home-based cancer patients reported that distress scores can be high in the absence of a caregiver [30].

In this study, Class 1, which was characterized by lack of a caregiver but presence of high self-care, had a low distress score. This may have been the case because it is possible to live without other caregivers due to the low level of severity on the problem list. Another previous study [29] reported that the distress score was at 5.50 when the primary caregiver was a parent; this finding was consistent with this study. However, the distress score was low at 3.42 when the primary caregiver was a spouse, which was inconsistent with this study. It is necessary to discern whether this difference was due to the age difference of the study participants rather than the difference in the identity of the primary caregiver, since young patients mostly live with their parents or siblings. As for clinical characteristics, there were differences in cancer type, time of diagnosis of cancer, and subjective health by class. Class 2 with greater number of problems had a higher rate of other cancers (gynecological cancer, thyroid cancer, stomach cancer, etc.) than Class 1 with low problems. Meanwhile, the number of breast cancer patients was high in Class 3, probably because this group included many young and unmarried female participants. Therefore, it is necessary to determine whether the difference in this problem list can be attributed to the type of cancer or other characteristics of the participants through future research that considers participants’ age, sex, and type of cancer. In terms of the time of diagnosis, 23.1% of cases in Class 1 been diagnosed for more than 5 years, but this was true for only 6.6% of cases in Class 2. In general, long-term survivors of more than five years are considered as “cured” from the prevailing medical point of view because they have overcome the physical and psychological burden caused by disease and treatment to some extent and have adapted to a new situation [31]. This suggests that it is important to actively implement follow-up management within five years. In this study, Class 3, which was identified as having the highest distress, had low family problems, and the highest emotional problems. However, there was no statistically significant difference in distress compared to Class 2, where all problems were high, so it is necessary to consider the characteristics of the participants rather than the severity of the problems. In Class 3, where one of the four problems was low, we expected that the distress level would be relatively lower than that of the group where all problems were high. However, it was not, and this was probably due to another cultural and situational stressor. Furthermore, in Class 3, all the participants were women, and many of them were unmarried and diagnosed with breast cancer. Since a large part of a woman’s identity is often socially tied to their breasts, they may experience a sense of loss and deprivation as women [32] and have higher emotional problems and distress.

The family is the primary environment surrounding an individual and can positively or negatively impact patients undergoing crises such as cancer diagnosis and treatment [33]. In this study, most participants were women, and it is therefore necessary to consider the position and role of women in the traditional Asian patriarchal family structure. For women, motherhood can signify ambivalent feelings of guilt regarding their children or a driving force that can strengthen their will to recover. Furthermore, depending on the role and responsibility of housewives, women may face difficulties and conflicts due to the demands for housework [32]. Therefore, it is necessary to re-examine the pattern of problem list clusters in other cultures while considering cultural differences. Moreover, psychological problems are caused by psychosocial factors rather than physical factors; family support, spouse support, and so on can greatly affect according to the characteristics of the Asian family structure [32]. This study confirmed the distress based on primary caregivers and marital status but did not confirm the level of family support. Further studies are necessary for determining whether there is a difference in family support between the groups in the problem list.

This study has several limitations. First, this study collected data from cancer survivors in certain regions and included a large number of women and patients who had been diagnosed with a specific cancer. Convenient sampling and cross-sectional design may limit the generalizability of these study results. It is necessary to recruit participants after considering gender and types of cancer, further analyze the health problems of cancer survivors, and collect nationwide data for generalizability. Second, there was no standard recommendation for conducting LPA with regard to problem list analysis of cancer patients or cancer survivor symptoms. We recommend that additional studies should compare research based on incidence and severity. Third, non-parametric analysis was performed to confirm the difference in results according to class due to the heteroscedasticity of the data.

Despite these limitations, this study is meaningful in that it identified differences in characteristics and distress by classifying survivors according to the severity of their problems. When developing or applying an intervention for distress, it is important to apply an integrated intervention rather than an intervention limited to one area. It is also necessary to develop a tailored intervention that considers a list of problems experienced by cancer survivors as well as cultural characteristics.

## 5. Conclusions

This study classified three latent classes using four indices: physical, emotional, functional, and family problems. There was a difference in the degree of distress based on the problem list profile. This result could contribute to knowledge about cancer survivors’ problem list and distress by using unique statistical analysis. Based on these results, healthcare professionals should provide group-specific interventions while considering participants’ characteristics to help cancer survivors deal with distress-related problems and improve their quality of life.

## Figures and Tables

**Table 1:** Demographic and Clinical Characteristics (*N* = 401).

Variables	Categories	*n* (%) or Mean ± SD
Age (years)		45.74 ± 8.14
Gender	Female	376 (93.8)
	Male	25 (6.2)
Education	≤High school	74 (18.5)
	College/university	283 (70.6)
	≥Graduate school	44 (11.0)
Marriage	Single	85 (21.2)
	Married	291 (72.6)
	Divorce/widowed	25 (6.2)
Major health caregiver	Spouse	151 (37.7)
	Parent	49 (12.2)
	Son/daughter	58 (14.5)
	Sibling	13 (3.2)
	Self-care	130 (32.4)
Employment status	Unemployed	258 (64.3)
	Employed	143 (35.7)
Monthly household income (10,000 won)	≤200	60 (15.0)
	200~499	172 (42.9)
	≥500	169 (42.1)
Place of residence	Capital area	290 (72.3)
	Non-capital area	111 (27.7)
Cancer types	Gastric cancer	31 (7.7)
	Thyroid cancer	33 (8.2)
	Colorectal cancer	12 (3.0)
	Breast cancer	254 (63.3)
	Gynecologic Cancer	42 (10.5)
	Others	29 (7.2)
Time of diagnosis of cancer (month)	<23	95 (23.7)
	24~59	250 (62.3)
	≥60	56 (14.0)
Period since last treatment (months)	0~11	141 (35.2)
	12~23	120 (29.9)
	24~35	840 (19.9)
	36~47	44 (11.0)
	48~60	16 (4.0)
Subjective health		2.69 ± 0.61

**Table 2:** Model Fit Indices of Latent Profile Analysis and Distribution Rate of Problem list (*N* = 401).

Model	Model Fit Indices					Latent Class Distribution Rate (%)			
	BIC	saBIC	LMR	BLRT	Entropy	1	2	3	4
1 profile	7229.707	7204.322	na	na	na	100			
2 profile	6753.529	6712.279	<0.001	<0.001	0.851	36.4	63.6		
3 profile	6668.667	6611.552	0.007	<0.001	0.876	36.7	49.1	14.2	
4 profile	6629.881	6556.901	0.093	<0.001	0.846	33.1	29.7	13.5	23.7

BIC = Bayesian Information Criteria; saBIC = sample-size adjusted BIC; LMR = Lo-Mendell-Rubin adjusted likelihood ratio test; BLRT = parametric bootstrapped likelihood ratio test; na = not applicable.

**Table 3:** Differences of Indices of Problem List Among Latent Classes (*N* = 401).

	Class	Class 1	Class 2	Class 3	Total	F (*p*) Post Hoc
		(*n* = 147)	(*n* = 197)	(*n* = 57)		
Group Indices		Mean ± SD	Mean ± SD	Mean ± SD		
Physical		2.94 ± 1.30	5.61 ± 1.25	6.03 ± 1.23	4.69 ± 1.84	224.83 (<0.001)
						1 < 2, 3
Change in appearance (hair loss, skin color)		2.96 ± 2.68	5.46 ± 2.20	5.67 ± 2.92	4.57 ± 2.77	
Diet (weight/intake change)		2.96 ± 2.35	5.39 ± 2.19	5.88 ± 2.21	4.57 ± 2.56	
Fatigue		3.94 ± 2.05	6.81 ± 1.71	7.42 ± 1.55	5.84 ± 2.33	
Indigestion (nausea)		1.88 ± 1.87	5.00 ± 2.44	4.63 ± 2.45	3.81 ± 2.68	
Memory/reduced concentration		3.39 ± 2.12	6.24 ± 1.94	6.89 ± 2.04	5.29 ± 2.49	
Pain		2.47 ± 1.88	5.15 ± 2.08	5.54 ± 2.20	4.22 ± 2.43	
Sleep disorder		3.05 ± 2.44	5.80 ± 2.23	6.44 ± 2.69	4.88 ± 2.76	
Numbness of limbs		2.84 ± 2.37	5.04 ± 2.50	5.79 ± 2.62	4.34 ± 2.73	
Emotional		3.14 ± 1.52	6.40 ± 1.23	6.95 ± 1.64	5.28 ± 2.16	272.47 (<0.001)
						1 < 2 < 3
Depression/sadness		2.53 ± 1.87	5.57 ± 1.89	6.33 ± 2.21	4.57 ± 2.48	
Fear/worry		3.51 ± 2.04	6.69 ± 1.85	7.47 ± 1.60	5.64 ± 2.50	
Nervousness/irritability		2.71 ± 1.91	6.16 ± 2.08	6.35 ± 2.50	4.92 ± 2.68	
Loss of motivation		2.59 ± 1.89	5.96 ± 2.03	6.23 ± 2.42	4.76 ± 2.63	
Anxiety about recurrence/death		3.87 ± 2.48	6.96 ± 1.92	7.60 ± 2.13	5.92 ± 2.68	
Worries about the meaning of life		3.64 ± 2.31	7.03 ± 1.95	7.70 ± 1.95	5.88 ± 2.70	
Functional		2.82 ± 1.99	5.86 ± 1.94	5.93 ± 2.35	4.76 ± 2.50	106.17 (<0.001)
						1 < 2, 3
Economic problems		3.16 ± 2.32	6.31 ± 2.16	6.11 ± 2.65	5.13 ± 2.73	
Work/school		2.49 ± 2.33	5.41 ± 2.52	5.75 ± 2.82	4.39 ± 2.88	
Family		1.22 ± 1.30	5.63 ± 1.51	0.50 ± 0.84	3.28 ± 2.68	586.05 (<0.001)
						3 < 1 < 2
Raising children		1.24 ± 1.52	6.24 ± 2.11	0.61 ± 1.41	3.61 ± 3.17	
Problems with children		1.17 ± 1.42	5.49 ± 1.98	0.33 ± 0.72	3.17 ± 2.83	
Relationship with spouse		1.24 ± 1.75	5.15 ± 2.42	0.54 ± 1.31	3.06 ± 2.92	

**Table 4:** Comparison of distress between the three classes (*N* = 401).

Variables	Total	Class 1	Class 2	Class 3	F (*p*) Post hoc
	(*n* = 401)	(*n* = 147)	(*n* = 197)	(*n* = 57)	
	M ± SD	M ± SD	M ± SD	M ± SD	
Distress	4.97 ± 2.17	3.33 ± 1.86	5.81 ± 1.59	6.28 ± 2.14	43.69 (<0.001)
					1 < 2 = 3

Adjusted for age, education, marriage, major health caregiver, income, cancer type, time of diagnosis of cancer, and subjective health (Non-parametric ANCOVA).

**Table 5:** Differences in the characteristics of participants according to the profile group (*N* = 401).

Characteristics	Categories	Total	Class 1	Class 2	Class 3	χ^2^ or F (*p*)
		*n* (%) or	*n* (%) or	*n* (%) or	*n* (%) or	
		M ± SD	M ± SD	M ± SD	M ± SD	
Age (years)		45.74 ± 8.14	47.61 ± 9.04	45.51 ± 6.62	41.70 ± 8.96	11.57 (<0.001)
						3 < 1, 2
	Below 30 s	98 (24.4)	34 (23.1)	41 (20.8)	23 (40.4)	27.21 (<0.001)
	40 s	208 (51.9)	62 (42.4)	120 (60.9)	26 (45.6)	
	50 s	72 (18.0)	37 (25.2)	30 (15.2)	5 (8.8)	
	Over 60 s	23 (5.7)	14 (9.5)	6 (3.0)	3 (5.3)	
Gender	Male	25 (6.2)	9 (6.1)	16 (8.1)	0 (0.0)	4.99 (0.082)
	Female	376 (93.8)	138 (93.9)	181 (91.9)	57 (100.0)	
Education level	≤High school	74 (18.5)	36 (24.5)	29 (14.7)	9 (15.8)	10.36 (0.035)
	College/university	283 (70.6)	92 (62.6)	152 (77.2)	39 (68.4)	
	≥Graduate school	44 (11)	19 (12.9)	16 (8.1)	9 (15.8)	
Marital status	Single	85 (21.2)	36 (24.5)	10 (5.1)	39 (68.4)	111.72 (<0.001)
	Married	291 (72.6)	99 (67.3)	177 (89.8)	15 (26.3)	
	Divorce/widowed	25 (6.2)	12 (8.2)	10 (5.1)	3 (5.3)	
Primary health caregiver	Spouse	151 (37.7)	55 (37.4)	85 (43.1)	11 (19.3)	119.07 (<0.001)
	Parent & Sibling	62 (15.5)	21 (14.3)	13 (6.6)	28 (49.1)	
	Children	58 (14.5)	2 (1.4)	54 (27.4)	2 (3.5)	
	Self-care	130 (32.4)	69 (46.9)	45 (22.8)	16 (28.1)	
Employment	Unemployed	258 (64.3)	88 (59.9)	138 (70.1)	32 (56.1)	5.75 (0.056)
status	Employed	143 (35.7)	59 (40.1)	59 (29.9)	25 (43.9)	
Monthly household income (10,000 won)	≤200	60 (15.0)	17 (11.6)	24 (12.2)	19 (33.3)	39.59 (<0.001)
	200~499	172 (42.9)	85 (57.8)	67 (34.0)	20 (35.1)	
	≥500	169 (42.1)	45 (30.6)	106 (53.8)	18 (31.6)	
Place of residence	Capital area	111 (27.7)	47 (32.0)	50 (25.4)	14 (24.6)	2.15 (0.341)
	Non-capital area	290 (72.3)	100 (68.0)	147 (74.6)	43 (75.4)	
Cancer types	Breast cancer	254 (63.3)	104 (70.7)	104 (52.8)	46 (80.7)	20.31 (<0.001)
	Others ^1^	147 (36.7)	43 (29.3)	93 (47.2)	11 (19.3)	
Time of diagnosis of cancer (years)	<2	95 (23.7)	38 (25.9)	43 (21.8)	14 (24.6)	23.01 (<0.001)
	2~5	250 (62.3)	75 (51.0)	141 (71.6)	34 (59.6)	
	≥5	56 (14.0)	34 (23.1)	13 (6.6)	9 (15.8)	
Period since last treatment (years)	<1	141 (35.2)	51 (34.7)	71 (36.0)	19 (33.3)	12.49 (0.131)
	1~2	120 (29.9)	35 (23.8)	67 (34.0)	18 (31.6)	
	2~3	80 (20.0)	36 (24.5)	36 (18.3)	8 (14.0)	
	3~4	44 (11.0)	16 (10.9)	20 (10.2)	8 (14.0)	
	4~5	16 (4.0)	9 (6.1)	3 (1.5)	4 (7.0)	
Subjective health		2.69 ± 0.61	3.03 ± 0.54	2.51 ± 0.55	2.42 ± 0.60	45.03 (<0.001)
						2, 3 < 1

^1^ 11 Types of cancer with 40 gynecologic cancer, 33 thyroid cancer, 31 stomach cancer and etc.

## Data Availability

The data presented in this study are available on request from the corresponding author.
